# Concurrent conventionally factionated radiotherapy and weekly docetaxel in the treatment of stage IIIb non-small-cell lung carcinoma

**DOI:** 10.1038/sj.bjc.6690599

**Published:** 1999-08

**Authors:** M I Koukourakis, N Bahlitzanakis, M Froudarakis, A Giatromanolaki, V Georgoulias, S Koumiotaki, M Christodoulou, G Kyrias, J Skarlatos, J Kostantelos, K Beroukas

**Affiliations:** Department of Radiotherapy and Oncology and Laboratory of Cancer Cell Biology, University Hospital of Heraklion, PO Box 1352, Crete, Greece; Department of Lung Disease, Venizelion General Hospital, Crete, Greece; Department of Radiotherapy and Oncology, Hellenic Cancer Institute, Saint Savvas Hospital, Athens, Greece

**Keywords:** docetaxel, radiotherapy, lung cancer

## Abstract

Docetaxel has shown remarkable radiosensitizing in vitro properties. In a previous phase I/II dose escalation study in non- small-cell lung cancer (NSCLC) we observed a high response rate after concomitant boost radiotherapy and weekly docetaxel. The maximum tolerated dose was 30 mg m^−2^ week^−1^. In the present phase II study we evaluated whether weekly docetaxel and conventionally fractionated radiotherapy could be better tolerated and equally effective in the treatment of locally advanced NSCLC. Thirty-five patients with T3, T4/N2, T3/M0-staged disease were recruited. Docetaxel (30 mg m^−2^) was given as a 30 min infusion once a week. Asthenia and radiation-induced oesophagitis were the main side-effects of the regimen enforcing 2-week treatment delay in 6/35 (17%) patients and minor delay (3–7 days) in another 11/35 (31%) patients. Neutrophil, platelet and haemoglobin toxicity was minimal, but pronounced lymphocytopenia was observed. Complete response (CR) of the chest disease was observed in 12/35 (34%) patients and partial response in 16/35 (46%). Although not statistically significant (*P* = 0.19), a higher CR rate (8/18; 44%) was observed in patients who accomplished their therapy within the scheduled treatment time (44–47 days) as compared to patients that interrupted their treatment for several days due to treatment-related toxicity (CR 4/17; 23%). The overall survival and the local progression-free survival at 1 year was 48% and 60% respectively. We conclude that docetaxel combination with radiotherapy is a promising approach for the management of locally advanced NSCLC that results in high CR rate. Further trials with docetaxel-based radiochemotherapy should integrate accelerated radiotherapy together with cytoprotection. © 1999 Cancer Research Campaign


*British Journal of Cancer* (1999) **80**(11), 1792–1796
© 1999 Cancer Research Campaign
Article no. bjoc.1999.0599

### Concurrent conventionally factionated radiotherapy and weekly docetaxel in the treatment of stage IIIb non-small-cell lung carcinoma

**MI Koukourakis[1], N Bahlitzanakis[2], M Froudarakis[1], A Giatromanolaki[1], V Georgoulias[1], S Koumiotaki[2], M Christodoulou[2], G Kyrias[3], J Skarlatos[3], J Kostantelos[3] and K Beroukas[3]**

[1]Department of Radiotherapy and Oncology and Laboratory of Cancer Cell Biology, University Hospital of Heraklion, Heraklion 71110, PO Box 1352, Crete, Greece; [2]Department of Lung Disease, Venizelion General Hospital, Heraklion, Crete, Greece; [3]Department of Radiotherapy and Oncology, Hellenic Cancer Institute, Saint Savvas Hospital, Athens, Greece

**Summary** Docetaxel has shown remarkable radiosensitizing in vitro properties. In a previous phase I/II dose escalation study in non-small-cell lung cancer (NSCLC) we observed a high response rate after concomitant boost radiotherapy and weekly docetaxel. The maximum tolerated dose was 30 mg $m^{-2}$ $week^{-1}$. In the present phase II study we evaluated whether weekly docetaxel and conventionally fractionated radiotherapy could be better tolerated and equally effective in the treatment of locally advanced NSCLC. Thirty-five patients with T3, T4/N2, T3/M0-staged disease were recruited. Docetaxel (30 mg $m^{-2}$) was given as a 30 min infusion once a week. Asthenia and radiation-induced oesophagitis were the main side-effects of the regimen enforcing 2-week treatment delay in 6/35 (17%) patients and minor delay (3–7 days) in another 11/35 (31%) patients. Neutrophil, platelet and haemoglobin toxicity was minimal, but pronounced lymphocytopenia was observed. Complete response (CR) of the chest disease was observed in 12/35 (34%) patients and partial response in 16/35 (46%). Although not statistically significant ($P = 0.19$), a higher CR rate (8/18; 44%) was observed in patients who accomplished their therapy within the scheduled treatment time (44–47 days) as compared to patients that interrupted their treatment for several days due to treatment-related toxicity (CR 4/17; 23%). The overall survival and the local progression-free survival at 1 year was 48% and 60% respectively. We conclude that docetaxel combination with radiotherapy is a promising approach for the management of locally advanced NSCLC that results in high CR rate. Further trials with docetaxel-based radiochemotherapy should integrate accelerated radiotherapy together with cytoprotection.

**Keywords:** docetaxel; radiotherapy; lung cancer

Although radiotherapy is effective in early lung cancer, 5-year overall survival in stage IIIb is less than 10% after radiotherapy (Koukourakis et al, 1995). The addition of chemotherapy before or concurrently with irradiation may slightly improve the survival, but discouraging results have been also reported by randomized trials (Mattson et al, 1988; Dillman et al, 1990; Morton et al, 1991; Le Chevalier et al, 1992; Brodin et al, 1996). Forty-five per cent of patients with locally advanced disease die from local recurrence without distant metastasis, which suggests that effective local therapy may prolong disease-free survival or even cure a subset of patients with non-metastasizing tumour phenotype (Koukourakis et al, 1995). An effective non-surgical regimen that would increase the response rate could be also useful in downstaging inoperable disease or even in improving survival of operable cases.

Recently, several novel agents with remarkable radiosensitizing properties have been introduced in clinical practice. Taxanes are inhibitors of microtubule depolymerization (Geuritte-Voegelein et al, 1991; Ringel et al, 1991). Radiation-sensitizing effects of docetaxel have been confirmed in vitro (Choy et al, 1992) and are probably related to the cell synchronization effect to the radio-sensitive G2/M cell cycle phases (Chaffey et al, 1971). Using the HL-60 cell line, the sensitizing enhancement ratio at low doses of docetaxel (0.03 μM), was 2.15 (Choy et al, 1992). In vitro data showing a putative role of taxanes in phosphorylation of the bcl-2 anti-apoptotic oncoprotein (Haldar et al, 1996) suggest that taxanes may further enhance the radiation efficacy by facilitating the switch-on of the apoptotic machinery after DNA damage by radiotherapy. Nuclear import of p53 protein after bcl-2 protein phosphorylation by paclitaxel resulted in induction of apoptosis in RKO cells treated with γ-radiation (Beham et al, 1998). Docetaxel has also shown remarkable response rates (23–33%) in phase II studies for advanced non-small-cell lung cancer (NSCLC) (Cerny et al, 1994; Fossella et al, 1994; Francis et al, 1994).

In a previous phase I/II study we established a well-tolerated regimen of weekly docetaxel administration together with slightly accelerated (5-week regimen) concomitant boost radiotherapy for NSCLC (Koukourakis et al, 1998). The maximum tolerated dose was 30 mg $m^{-2}$ $week^{-1}$. An increased incidence of oesophagitis and of asthenia was observed, but the encouraging high response rate (77%) justified further clinical investigation. In the present study we sought to confirm the high complete response (CR) rate previously observed in a phase I trial in a new cohort of patients with locally advanced non-metastatic NSCLC. We made the hypothesis that the cell synchronization effect of docetaxel may abrogate the phenomenon of rapid tumour repopulation during radiotherapy so

*Received 24 August 1998*
*Revised 25 January 1999*
*Accepted 20 February 1999*

*Correspondence to*: MI Koukourakis, Tumour and Angiogenesis Research Group, 18 Dimokratias Avenue, Iraklion 71306, Crete, Greece

**Table 1** Patient characteristics.

| | |
|---|---|
| Total no. of patients | 35 |
| Male:female | 33:2 |
| Age, years | |
| Median | 64 |
| Range | 45–78 |
| WHO PS | |
| Median | 1 |
| Range | 0–2 |
| TNM-stage | |
| T3,4/N2/M0 | 10 |
| T3/N3/M0 | 20 |
| T4/N3/M0 | 5 |
| Prior treatment | |
| Chemotherapy naive | 20 |
| Taxane chemotherapy | 6 |
| Platinum chemotherapy | 10 |
| Tumour type | |
| Squamous cell | 21 |
| Adenocarcinoma | 9 |
| Undifferentiated | 5 |

that acceleration of radiotherapy could become less important in the outcome of radiotherapy (Koukourakis et al, 1996). Conventionally fractionated radiotherapy was therefore used in this phase II study in order to evaluate whether weekly docetaxel and non-accelerated radiotherapy could be better tolerated and equally effective in the treatment of locally advanced NSCLC.

### PATIENTS AND METHODS

### Recruitment criteria

Thirty five patients with histologically confirmed stage T3, T4/N2, T3 (TNM; Hermanek and Sobin, 1992) NSCLC entered this phase II study. Patients should have a PS < 3. Written informed consent was obtained from all patients. Patients previously treated with radiotherapy to another than chest site or chemotherapy (including paclitaxel or docetaxel), completed at least 2 months before recruitment were also eligible. Patients with known reduced pulmonary reserve (FEV1 < 800 mL, $pO_2$ < 60, $pCO_2$ > 45), related to chronic obstructive lung diseases, were excluded from the study. However, patients with impaired pulmonary reserve caused by the tumour itself were eligible. Patients with white blood cells < 2500 $\mu l^{-1}$ and platelets (Pt) < 120 000 $\mu l^{-1}$ were excluded. Patients with haemoglobin (Hb) < 10 g $ml^{-1}$ were transfused until Hb levels raised > 11 g $dl^{-1}$. Pregnant women or patients with major heart, liver, renal, psychiatric disease or haematological malignancies were also excluded. Patients with known episodes of collapsus as a result of allergic response to any drug or substance were excluded. Table 1 shows the patient characteristics.

### Pretreatment and treatment evaluation

Baseline studies included physical examination, chest X-rays, whole blood count (WBC) with differential and platelet count, complete biochemical profile, bone scan and computerized tomography (CT) of the chest and upper abdomen. Patients were followed with WBC, serum urea, and creatinine and liver enzymes once a week during the radiotherapy period and for 4 weeks thereafter. Chest X-ray and electrocardiogram (ECG) were performed every 2 weeks. Acute radiation toxicity was registered twice weekly and radiotherapy delay was enforced in case of grade 3 diarrhoea, cystitis or oesophagitis. The WHO scale (World Health Organization, 1979) was used to assess chemotherapy and acute radiation toxicity.

Response to treatment was assessed with CT scan of the chest lesion on day 25 (to allow eventual modification of the radiotherapy fields) and 45–60 days after treatment completion. Duration of response was measured from the time the criteria of the objective response were first met with CT scan done every 2 months for the first 6 months, and every 3–4 months (or earlier if necessary) thereafter. CR was defined as 95–100% reduction of the chest measurable lesion within 2 months after treatment completion. Given the difficulties of assessing response to radiotherapy of large masses, any residual scar measuring less than 5% of the initial tumour volume that did not progress for at least 2 months following response documentation was still considered as complete response. Similarly, partial and minimal response refers to 50–95% and 25–49% reduction of tumour dimensions respectively. Small reduction of tumour dimensions between 0 and 24% that lasted at least 2 months after response documentation were considered as stable disease. All other cases were considered as progressive disease regardless of the initial response.

### Radiotherapy schedule

Radiotherapy treatment planning was based on recent chest CT scan. Antero-posterior portals encompassing the primary tumour and part of the mediastinum were used to deliver a daily dose of 2 Gy (five fractions per week) to a total dose of 44 Gy. The mean dimensions of these large portals were 264 $cm^2$ (range 210–355 $cm^2$). Homolateral supraclavicular area was included in cases with an upper lobe mass. One or two oblique fields limited to the bulky tumoral area (2 Gy per fraction) were used to increase the total tumour dose to 64 Gy. The mean dimensions of the booster fields were 51 $cm^2$ (range 29–124 $cm^2$). Patients with pleural effusion, lung atelectasia or very large tumours were irradiated to the whole hemithorax using anteroposterior fields, 1.5 Gy daily to a total dose of 18 Gy. The planned overall treatment time was 6.5 weeks (44 days).

### Docetaxel administration

Twelve hours and 30 min before chemotherapy patients received 32 mg oral (p.o.) and 125 mg intravenous (i.v.) bolus methyl-prednisolone respectively. Ranitidine 300 mg p.o. was given daily throughout the 6-week treatment. Docetaxel was diluted in 250 ml normal saline and infused within 20 min. Tropisetron (5 mg i.v.) was given as anti-emetic treatment. Blood pressure and symptomatology assessment were monitored every 5 min during infusion, and every 15 min for the following hour. No steroids were used thereafter if no allergic reaction occurred. Whenever allergic reaction was observed patients were given methyl-prednisolone (32 mg p.o.) 12 h after chemotherapy.

The docetaxel level was 30 mg $m^{-2}$ $week^{-1}$. Six-weekly cycles of the drug were to be delivered during the 6-week course of radiotherapy. Neutrophil grade 2 toxicity was to be adjusted with granulocyte colony-stimulating factor (G-CSF) administration (300 $\mu g$ $m^{-2}$ subcutaneously (s.c.) on Saturday and Sunday every week) starting immediately after diagnosis and continuing throughout the radiotherapy period. Our previous experience with

© 1999 Cancer Research Campaign

**Table 2** Haematological, non-haematological toxicity and radiotherapy delay in 35 patients with stage IIIb non-small cell lung cancer treated with standard fractionation of radiotherapy and weekly docetaxel (30 mg $m^{-2}$) chemotherapy

| | Grade | | | | RT delay[a] | |
|---|---|---|---|---|---|---|
| | 0/1 | 2 | 3 | 4 | ≤ 7 days | > 7 days |
| Anorexia | 35 | 0 | 0 | 0 | 0 | 0 |
| Asthenia | 27 | 8 | 0 | 0 | 5 | 3 |
| Alopecia | 33 | 2 | 0 | 0 | 0 | 0 |
| Fever | 30 | 5 | 0 | 0 | 5 | 0 |
| Hot-flushes | 11 | 0 | 0 | 0 | 0 | 0 |
| Oesophagitis | 18 | 5 | 12 | 0 | 8 | 6 |
| Fungal infection | 32 | 3 | 0 | 0 | 0 | 3 |
| Cough | 21 | 14 | 0 | 0 | 0 | 0 |
| Radiation pneumonitis | 27 | 4 | 3 | 1 | (late toxicity) | |
| Hypotenstion | 32 | 3 | 0 | 0 | 0 | 0 |
| Leg oedema | 33 | 2 | 0 | 0 | 0 | 0 |
| Pleural effusion | 32 | 3 | 0 | 0 | 0 | 0 |
| Neurosensory | 4 | 0 | 0 | 0 | 0 | 0 |
| Neutropenia | 3 | 2 | 0 | 0 | 0 | 0 |
| Haemoglobin | 35 | 0 | 0 | 0 | 0 | 0 |
| Platelets | 35 | 0 | 0 | 0 | 0 | 0 |
| Lymphocytes | 0 | 0 | 5 | 30 | 0 | 0 |

[a] Number of patients for which the reported toxicity significantly contributed to the enforcement of radiotherapy delay.

weekly docetaxel during concomitant boost radiotherapy shows that the expected haematological toxicity of the docetaxel schedule usedin the present study is minimal. However, in order to avoid delays of chemotherapy administration or undesired dose reductions, a low dose G-CSF schedule was included in the initial design ofthe study for the rare cases that would present with grade 2 neutropenia. The effectiveness of this low-dose G-CSF schedule in preventing neutropenia during fractionated chemoradiotherapy has been established in a previous study of ours (Koukourakis et al, 1999). Radiotherapy or chemotherapy was not interrupted unless progression of neutropenia appeared. Grade 3/4 toxicity was to be followed by 50% dose reduction or chemotherapy interruption, depending upon severity.

### Statistical analysis

The statistical analysis and graph presentation of survival curves was performed using the GraphPad Prism 2.01 version package. Statistics between variables were performed using the Fisher's exact test or the paired two-tailed *t*-test, as appropriate. Survival curves were plotted using the method of Kaplan and Meier. $P$ values < 0.05 were considered to be statistically significant.

### RESULTS

### Non haematological docetaxel-related toxicity

Severe hypersensitivity reactions during docetaxel infusion were seen in 2/35 patients leading to the interruption of the infusion. Methyl-prednizolone 250 mg i.v. were immediately given. Both patients received their treatment 30 min later with no further complications. Hot flushes were observed in 11/35 (31%) cases.

Steroid-related toxicity was minimal since the high dose of methyl-prednizolone was restricted to 1 day per week.

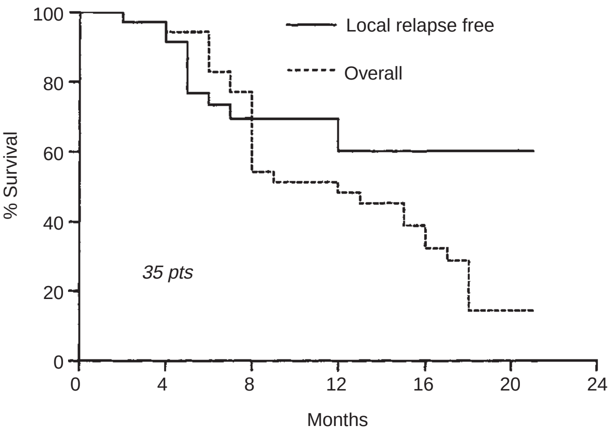

**Figure 1** Kaplan–Meier survival curves in 35 patients with stage IIIb non-small-cell lung carcinoma treated with conventionally fractionated radiotherapy and weekly 30 mg $m^{-2}$ of docetaxel

Prophylactic administration of ranitidine 300 mg daily was given for 45 days, starting from the beginning of treatment. Transient increase in blood glucose levels never required the use of insulin.

Table 2 shows the non-haematological toxicity. Neurosensory toxicity grade 1 was observed in 4/35 (11%) patients during the 3rd–5th cycle and regressed 2–4 weeks after therapy. Mild asthenia was constantly observed. Asthenia was the only reason for a 1-week treatment delay in 5/35 (17%) patients. Asthenia, together with grade 2 oesophagitis, was the reason of a 2-week treatment delay in 3/35 (8.5%) patients. Anorexia and taste alteration were observed in all 35 patients and were the only side-effects of therapy in 3/35 (8%) patients. The mean weight loss was 4 kg (range 3–6). Grade 1 alopecia was observed in 25/35 (71%) and grade 2 in 2/35 (6%) patients. Fever up to 39°C (grade 2), without confirmation of infection, was observed in 5/35 (14%) patients during the 3rd–4th week. Omission of docetaxel for 1 week resulted in normalization of the body temperature within 3–5 days. Grade 2 hypotension was observed in 3/35 patients (8%). Three patients (8.5%) developed bilateral grade 2 leg oedema and 3/35 (8.5%) pleural effusion during the 4th week of therapy. Pleural effusion was treated with furosemide and spirolactone and was resolved within 4–8 weeks after therapy. Hot flushes were seen in 5/35 (14%) patients. No headache, arthralgia-myalgia, nausea-vomiting, diarrhoea, mucositis or nail disorders were observed.

### 'In-field' radiotherapy-related toxicity

Radiation-induced grade 3 oesophagitis that resulted in a 1-week treatment delay was observed in 6/35 patients (17%). Two-week treatment delay due to severe oesophagitis was necessary in another 6/35 (17%) patients, where pharyngo-oesophageal candidiasis was also confirmed in three patients. In 5/6 patients the oesophagitis started during the 3rd–4th week of treatment and regressed within 1 week following treatment interruption. However, grade 3 oesophagitis recurred during the 5th–6th week of therapy enforcing further treatment delay. Patients were treated with anti-fungal p.o. therapy and analgesics. 'In-field' grade 2 radiation skin toxicity was observed in 2/35 (4%) patients.

Twenty eight cases completed 8–21 months of follow-up. One patient (3.7%) died from radiation pneumonitis 4 months after

© 1999 Cancer Research Campaign

**Table 3** Response rate after 64 Gy of standard fractionation of radiotherapy given together with weekly docetaxel, according to overall treatment time

| Overall treatment time (days) | No. of patients | CR (%) | PR (%) | NR (%) | *P*-value |
|---|---|---|---|---|---|
| 44–47 | 18 | 8 (44) | 7 (39) | 3 (17) | |
| 50–54 | 11 | 3 (27) | 7 (63) | 1 (10) | >0.12 |
| 57–61 | 6 | 1 (17) | 2 (33) | 3 (50) | |

radiotherapy. Seven out of 28 (25%) developed localized pulmonary fibrosis. Mild exertional dyspnoea was present in 3/7 patients, but there was no need for oxygen support in any of the patients ($pO^2$ > 60 mmHg). No patient developed radiation-related neurological or cardiac late toxicity.

### Haematological toxicity

Haemoglobin and neutrophil toxicity was minimal in all cohorts (Table 2). Grade 2 neutropenia was observed in 2/35 patients (5.7%) during the 4th week of therapy and received G-CSF prophylactically (5 μg $kg^{-1}$ $day^{-1}$ s.c. for 4 days). No platelet toxicity was observed. Severe lymphocytopenia was observed: the lymphocyte counts dropped from 1230 ± 532 $dl^{-1}$ down to 498 ± 213 $dl^{-1}$ during the 4th week ($P$ = 0.0003). Monocyte counts were also decreased from 567 ± 211 $dl^{-1}$ to 198 ± 120 $dl^{-1}$ ($P$ = 0.002).

### Response and survival

Complete response of the chest disease was observed in 12/35 (34%) patients and partial response in 16/35 (46%). The overall response rate was 80% (95% confidence interval (CI) 55–88%). Minimal response was observed in 4/35 (11%) patients and stable disease in the remaining 3/35 (8.5%).

We further analysed the CR rate in three groups of patients according to the overall treatment time (Table 3). Eight out of 18 (44%) patients that did not interrupt their treatment had a CR vs 3/11 (27%) and 1/6 (17%) of patients that interrupted their treatment for 1 and 2 weeks respectively. Although the difference was not statistically significant ($P$ = 0.19), there was a trend of a shorter overall treatment time to associate with a higher CR rate.

Eight out of 35 (23%) patients are alive with no evidence of disease 13–23 months after therapy. Twelve out of 35 died from local progression (five with distant metastasis), 14/35 from distant metastases (without evidence of local relapse) and one from radiation-induced pneumonitis. The Kaplan–Meier survival curve is shown in Figure 1. The median overall survival time was 12 months. The overall survival and the local progression-free survival at 1 year was 48% and 60% respectively.

### DISCUSSION

Docetaxel has shown substantial activity against NSCLC (Cerny et al, 1994; Fossella et al, 1994; Francis et al, 1994). In a previous phase I/II study we established a well-tolerated docetaxel scheme that could be administered concurrently with radiotherapy in patients with NSCLC (Koukourakis et al, 1998). Weekly doses up to 30 mg $m^{-2}$ were well-tolerated with minimal haematological toxicity and without severe asthenia. This regimen delivers a total dose of 90 mg $m^{-2}$ within 3 weeks, which is close to the maximum tolerated dose of docetaxel given as monotherapy (Tomiak et al, 1994). In a recent study by Maner et al (1998) a lower dose of docetaxel (20 mg $m^{-2}$) administered weekly with concomitant chest radiotherapy was suggested for phase II trials. However, in our phase I/II study the dose of 30 mg $m^{-2}$ weekly, together with concomitant boost radiotherapy, was well-tolerated. The high incidence of radiation-induced oesophagitis was not considered to be a major problem for this dose level to be tested in subsequent phase II trials. The 27% CR rate observed further encouraged the conducting of a pure phase II study.

In order to reduce the high rate of oesophagitis the concomitant boost-accelerated radiotherapy technique was replaced by a standard fractionation 6-week regimen. Thirty-five patients with stage IIIb NSCLC were enrolled in the study. The main toxicity observed was oesophagitis, which enforced a 2-week treatment delay in 6/35 (17%) patients and a minor treatment delay of 3–7 days in another 8/35 (23%) patients. Asthenia was frequent and often coincided with the onset of oesophagitis. In a previous study of docetaxel combination with accelerated radiotherapy (5-week regimen) (Koukourakis et al, 1998) the incidence of asthenia and oesophagitis was similar to the one observed in the present study, showing that standard fractionation does not improve the tolerability of the regimen. Complete response of the chest disease was observed in 12/35 (34%) patients and partial response in 16/35 (46%), which are similar to previously reported results (Koukourakis et al, 1998).

In a previous study we showed that the local control of disease and survival of patients with NSCLC depends on the overall treatment time (Koukourakis et al, 1996). We observed that in locally advanced tumours if the radiotherapy schedule extends beyond 35 days, 0.75 Gy from each radiotherapy fraction is consumed to compensate for rapid tumour repopulation. In the present study we made the hypothesis that docetaxel may abrogate the role of overall treatment time. However, despite the small number of cases, there was a trend for the CR to be more frequently observed in patients who accomplished their treatment without interruption (44 days). It could be therefore suggested that further trials with docetaxel-based radiochemotherapy should take into account the adverse effect of prolonged radiotherapy treatment time. Accelerated radiotherapy combination with docetaxel may result in higher CR rate. Given the high mucosal toxicity and asthenia observed, support with cytoprotective agents such as amifostin (Koukourakis, 1998), or even granulocyte–macrophage CSF (Throuvalas et al, 1995) may prove of importance in the feasibility of such a regimen.

The survival figures obtained in the present study are encouraging. The 48% overall survival and the 60% local progression-free survival at 1 year are similar to the results reported in a randomized trial of radiotherapy with concurrent daily *cis*-platinum (54% and 59% respectively) (Shaake-Koning et al, 1992). Our previous experience with radiotherapy alone shows that the 1-year survival in stage IIIb NSCLC is lower than 20% (Koukourakis et al, 1995, 1996). It should be stressed that all patients recruited in the present study had large tumoural burden and 15/35 (43%) of them had disease unresponsive to previous chemotherapy.

We conclude that docetaxel combination with radiotherapy is a promising approach for the management of locally advanced NSCLC. The high complete response rate observed encourages the use of this combination as a preoperative regimen in stage

*© 1999 Cancer Research Campaign*

IIb/IIIa. It is also anticipated that about 70% of patients with stage IIIb disease will show substantial reduction of the tumour burden, which may be important in the re-evaluation of the disease operability. Further investigation is required to assess the feasibility and efficacy of docetaxel combination with accelerated and/or hyperfractionated radiotherapy schedules supported with cytoprotection.

### REFERENCES

Beham M, Vogel M, McDonnell J, Farkas St., Furst A and Jauch KW (1998) Apoptosis after genotoxic damage is enhanced by Taxol-induced phosphorylation of bcl-2 and correlates with nuclear import of p53. *Proc Am Assoc Cancer Res* **39**: 463 (abstract no. 3151)

Brodin O, Nou E, Mercke C, Lindén CJ, Lundström R, Arwidi A, Brink J and Ringborg U (1996) Comparison of induction chemotherapy before radiotherapy with radiotherapy only in patients with locally advanced squamous cell carcinoma of the lung. The Swedish Lung Cancer Study Group. *Eur J Cancer* **32A**: 1893–1900

Cerny T, Kaplan S, Pavlidis N, Schöffski P, Epelbaum R, van Meerbeek J, Wanders J, Franklin HR and Kaye S (1994) Docetaxel (taxotere @) is active in non-small cell lung cancer: a phase II trial of EORTC Early Clinical Trials Group (ECTG). *Br J Cancer* **70**: 384–387

Chaffey JT and Hellman S (1971) Differing responses to radiation of murin bone marrow stem cells in relation to the cell cycle. *Cancer Res* **31**: 1513–1516

Choy H, Rodriguez F, Koester S, Hilsenbeck S and Von Hoff DD (1992) Synergistic effects of Taxol/Taxotere on radiation sensitivity on human tumor cell. *Int J Radiat Oncol Biol Phys* **24**: 274–275 (abstract no. 1059)

Dillman RO, Seagren SL, Propert KJ, Guerra J, Eaton WL, Perry MC, Carey RW, Frei EF III and Green MR (1990) A randomized trial of induction chemotherapy plus high-dose radiation versus radiation alone in stage III non-small-cell lung cancer. *N Engl J Med* **323**: 14940–14945

Fossella FV, Lee JS, Murphy WK, Lipman SM, Calayag M, Pang A, Chasen M, Shin DM, Glisson B, Benner S, Huber M, Perez-Soler R, Hong WK and Rober M (1994) Phase II study of docetaxel for recurrent or metastatic non-small cell lung cancer. *J Clin Oncol* **12**: 1238–1244

Francis P, Schneider J, Hann L, Bolmeceda C, Barakat R, Phillips M and Hakes T (1994) Phase II trial of docetaxel in patients with platinum-refractory advanced ovarian cancer. *J Clin Oncol* **12**: 2301–2308

Geuritte-Voegelein F., Guenard D, Lavelle F, Le Goff M, Mangatal L and Pottier P (1996) Relationships between the structure of Taxol analogues and their antimitotic activity. *J Med Chem* **34**: 992–998.

Haldar S, Chintapalli J and Croce CM (1996) Taxol induces bcl-2 phosphorylation and death of prostate cancer cells. *Cancer Res* **56**: 1253–1255

Hermanek P and Sobin J (1992) *UICC TNM Classification of Malignant Tumours*, 4th ed. Springer-Verlag: Berlin

Koukourakis M (1998) The use of ethyol in chemotherapy and radiation therapy. In: *Cytoprotection in Cancer Therapy: Improving the Therapeutic Index of Cancer Treatment*. Ethyol Investigators Meeting, May 1998, Los Angeles, California, USA.

Koukourakis M, Skarlatos J, Kosma L, Giatromanolaki A and Yannakakis D (1995) Radiotherapy alone for non-small cell lung carcinoma. Five year disease-free survival and patterns of failure. *Acta Oncol* **34**: 525–530

Koukourakis M, Hlouverakis G, Kosma L, Skarlatos J, Damilakis J, Giatromanolaki A and Yonnakakis D (1996) The impact of overall treatment time on the results of radiotherapy for non-small cell lung cancer. *Int J Radiat Oncol Biol Phys* **34**: 315–322

Koukourakis M, Kourousis C, Kamilaki M, Koukouraki S, Giatromanolaki A, Kakolyris S, Kotsakis A, Androulakis N, Bahlitzanakis N and Georgoulias V (1998) Weekly Docetaxel and Concomitant Boost Radiotherapy for Non-Small-Cell Lung Cancer. A Phase I/II Dose Escalation Trial. *Eur J Cancer* **34**: 838–844

Koukourakis M, Stefanaki E, Giatromanolaki A, Armenaki A, Fragiadaki C, Georgoulias V, Koumandakis E, Kranidis A and Helidonis E (1998) Fractionated carboplatin radiosensitization. A phase I dose escalation study. *Am J Clin Oncol* 1999 (in press)

Le Chevalier T, Arriagada R, Tarayre M, Lacombe-Terrier MJ, Laplanche A, Quoix E, Ruffie P, Martin M and Douillard JY (1992) Significant effect of adjuvant chemotherapy on survival in locally advanced non-small-cell lung carcinoma. *J Natl Cancer Inst* **84**: 58

Mattson K, Holsti LR, Holsti P, Jakobsson M, Kajanti M, Liippo K, Mäntylä M, Niitamo-Korhonen S, Nikkanen V and Nordman E (1998) Inoperable non-small cell lung cancer: radiation with or without chemotherapy. *Eur J Cancer Clin Oncol* **24**: 477–482

Mauers AM, Masters G, Haraf D, Hoffman PC, Watson SM, Golomb HM and Vokes EE (1998) Phase I study of docetaxel with concommitant thoracic radiotherapy. *J Clin Oncol* **16**: 159–164

Morton RF, Jett JR, McGinnis WL, Earle JD, Therneau TM, Krook JE, Elliott TE, Mailliard JA, Nelimark RA, Maksymiuk AW, et al (1991) Thoracic radiation therapy alone compared with combined chemoradiotherapy for locally unresectable non-small cell lung cancer. A randomized, phase III trial. *Ann Intern Med* **115**: 681–686

Ringel I and Horwitz SB (1991) Studies with RP-56976 (Taxotere): a semi-synthetic analogue of Taxol. *J Natl Cancer Inst* **83**: 288–291

Shaake-Koning C, van-den-Bogaert W, Dalesio O, Festen J, Hoogenhout J, van Houtte P, Kirkpatrick A, Koolen M, Maat B and Nijs A (1992) Effects of concomitant cisplatin and radiotherapy on inoperable non-small cell lung cancer. *N Engl J Med* **326**: 524–530

Throuvalas N, Antonadou D, Pulizzi M and Sarris G (1995) Evaluation of the efficacy and safety of GM-CSF in the prophylaxis of mucositis in patients with head and neck cancer treated by RT. In: *Proceedings of ECCO 1995*, pp. 593. Federation of European Cancer Societies: Paris (abstract 431).

Tomiak E, Piccart MJ, Kerger S, Lips S, Awada A, de Valeriola D, Lossignol D, Sculier JP, Auzannet V, Le Bail N, Bayssas M and Klastersky J (1994) Phase I study of docetaxel administered as 1-hour intravenous infusion on a weekly basis. *J Clin Oncol* **12**: 1458–1467

World Health Organization (1979) *Handbook for Reporting Results of Cancer Treatment*. WHO Offset Publications: Geneva.

 *© 1999 Cancer Research Campaign*
